# Disrupting interaction between miR-132 and *Mmp9* 3′UTR improves synaptic plasticity and memory in mice

**DOI:** 10.3389/fnmol.2022.924534

**Published:** 2022-08-05

**Authors:** Bozena Kuzniewska, Karolina Rejmak, Agata Nowacka, Magdalena Ziółkowska, Jacek Milek, Marta Magnowska, Jakub Gruchota, Olga Gewartowska, Ewa Borsuk, Ahmad Salamian, Andrzej Dziembowski, Kasia Radwanska, Magdalena Dziembowska

**Affiliations:** ^1^Laboratory of Molecular Basis of Synaptic Plasticity, Centre of New Technologies, University of Warsaw, Warsaw, Poland; ^2^Laboratory of Molecular Basis of Behavior, Nencki Institute of Experimental Biology of Polish Academy of Sciences, Warsaw, Poland; ^3^Laboratory of RNA Biology, International Institute of Molecular and Cell Biology, Warsaw, Poland; ^4^Faculty of Biology, University of Warsaw, Warsaw, Poland

**Keywords:** microRNA, miR132, *Mmp9*, synaptic plasticity, behavior, brain

## Abstract

As microRNAs have emerged to be important regulators of molecular events occurring at the synapses, the new questions about their regulatory effect on the behavior have araised. In the present study, we show for the first time that the dysregulated specific targeting of miR132 to *Mmp9* mRNA in the mouse brain results in the increased level of Mmp9 protein, which affects synaptic plasticity and has an effect on memory formation. Our data points at the importance of complex and precise regulation of the Mmp9 level by miR132 in the brain.

## Introduction

MicroRNAs (miRNAs) are important regulators of gene expression. These small, non-coding RNAs target mRNAs mainly through base pairing between the miRNA seed region and partially complementary sites in the 3′UTR (untranslated region) of the target mRNA. Such binding of miRNA to its target leads to translational repression and accelerated mRNA decay (Naeli et al., 2022).

Although cell culture models can provide mechanistic insight into the role of miRNAs in the regulation of the physiology of neurons, only animal models allow for understanding the impact of miRNA-mediated regulation at the organismal level. Animal models with knockdown or overexpression of genes encoding for selected miRNAs provided a valuable approach to study the role of miRNAs, primarily allowing identification of potential miRNA target genes *in vivo*. Such functional studies demonstrated the profound role of miRNAs in the regulation of brain development, physiology and animal behavior and suggested the contribution of miRNAs to neuropsychiatric and neurological disorders (Brennan and Henshall, 2020; Narayanan and Schratt, 2020). Still, one should keep in mind that miRNAs can exist singly or as clusters of different miRNA sequences, and a single miRNA can simultaneously regulate hundreds of mRNA target transcripts. The median number of potential targets for a given neuronal miRNA in the hippocampus was estimated as 500, ranging from 30 to more than 1,000 (Sambandan et al., 2017). Thus, targeted knock-out of one selected miRNA or even the whole miRNA cluster will have obvious implications for the dysregulation of hundreds of mRNA targets. Because of that, it is difficult to dissect the physiological role of specific miRNA-target interactions in the regulation of biological processes *in vivo*.

MicroRNA 132 (miR-132) is an experience-induced microRNA that is rapidly upregulated in model conditions of the synaptic plasticity, e.g., in the mouse primary visual cortex after eye-opening (Mellios et al., 2011), during long-term potentiation (LTP) in the rat hippocampus or upon brain-derived neurotrophic factor stimulation of primary cortical mouse neurons (Remenyi et al., 2010). MiR132 regulates dendritic arborization and neurite outgrowth during the development of hippocampal neurons and modulates dendritic spine morphology and synaptic transmission (Magill et al., 2010; Vo et al., 2010; Siegel et al., 2011; Wanet et al., 2012; Remenyi et al., 2013).

Matrix metalloproteinase 9 (Mmp9) is an endopeptidase involved in extracellular matrix remodeling. In the brain, Mmp9 plays a pivotal role in the regulation of dendritic spine morphology, synaptic plasticity, and learning and memory (Rivera et al., 2010; Michaluk et al., 2011; Huntley, 2012). Mmp9 expression, as well as its local translation at synapses, is induced by neuronal stimulation (Szklarczyk et al., 2002; Dziembowska et al., 2012).

Our previous study provided evidence for a direct link between those two essential regulators of synaptic plasticity: miR132 and Mmp9. We have shown that miR132 regulate the structural plasticity of dendritic spines through interaction with the *Mmp9* mRNA 3′UTR in primary hippocampal neurons (Jasinska et al., 2016).

The most reliable approach for confirming particular miRNA-target interaction and investigating its importance is to disrupt the miRNA-binding site within the endogenous target gene. Several attempts have been made to mutate miRNA binding sites *in vivo*, primarily using *Caenorhabditis elegans* as a model organism (Ecsedi et al., 2015; McJunkin and Ambros, 2017). Additionally, three mouse models with disrupted seed-match sequences in 3′UTRs of Socs1, Aicda, and Cdkn1b genes were created to study the role of miR155 and miR142 in the immune system (Dorsett et al., 2008; Lu et al., 2015; Mildner et al., 2017). However, there have been no reports of studying specific miRNA-target interaction *in vivo* in the mouse brain so far.

In the current manuscript, we elucidated the physiological consequences of miR132-*Mmp9* mRNA interaction *in vivo*. To address this question, we created a new knock-in mouse model with a disrupted miR132 binding site in *Mmp9* mRNA 3′UTR (*Mmp9* UTRmut). This approach allowed us to examine the implications of the resulting de-repression of the target mRNA at the organismal level. We detected elevated levels of Mmp9 protein in the hippocampus of *Mmp9* UTRmut mice, but the miR132 and *Mmp9* mRNA expression was not changed in this brain structure in course of development of the mutant mice. The *Mmp9* UTRmut mice displayed altered morphology of dendritic spines, which were more dense and shorter. Moreover, the hippocampal CA3-CA1 LTP was enhanced in *Mmp9* UTRmut mice. All the described changes in *Mmp9* UTRmut mouse neurons physiology impacted animal behavior since the mice exhibited enhanced learning in the hippocampus-dependent contextual fear-conditioning paradigm. In aggregate, in the present study, we show that an increased level of Mmp9 in the hippocampus resulting from lack of posttranscriptional regulation by miR132 affects the morphology of dendritic spines, synaptic plasticity and has an effect on memory formation in mice.

## Materials and methods

### Generating mouse model harboring mutation in *Mmp9* 3′UTR

A new mouse line B6.CBA *Mmp9*^em1Iimcb^/Tar (‘‘*Mmp9* UTRmut’’) was generated by the Mouse Genome Engineering Facility.^1^ All mice were bred and maintained in the animal house of Faculty of Biology, University of Warsaw under a 12-h light/dark cycle with food and water available *ad libitum*. The animals were treated in accordance with the EU Directive 2010/63/EU for animal experiments.

Basing on Mouse genome (GRCm38/mm10 Assembly) single guide RNA (sgRNA) was designed using Online CRISPR tool^2^ Chosen sequence did not show any major off-targets while calculated efficiency was high. For sgRNA synthesis two oligodeoxynucleotides (ODN) carrying T7 polymerase promoter, guide sequence (Mmp9_UTR_sgRNA_f) and sgRNA scaffold (Univ_IVsgRNA_rev) were used to form dsDNA template for *in vitro* transcription using in-house T7 Polymerase.

In a survey, 120 bp oligonucleotide Mmp9_UTRmut_O1 carrying point mutations to replace the wild-type miR132 seed match site in *Mmp9* 3′UTR was designed to introduce a unique. *Hpa*I (*Ksp*AI) restriction site for rapid and cost-effective genotyping. Cas9 mRNA was *in vitro* transcribed from Addgene pX458 plasmid using T7 RNA Polymerase, poly(A) tail and m7Gppp5′N Cap were added.

Injection cocktail (20 ng/μl Mmp9_UTR sgRNA IVT, 40 ng/μl Cas9 mRNA IVT, 50 ng/μl Mmp9_UTRmut_O1) was introduced into mice zygotes *via* microinjections. After 24–48 h of incubation embryos were implanted into surrogate mice.

### Genotyping

Pups were genotyped at age of 4 weeks. DNA from tail or ear tips was isolated with Genomic Mini kit (A&A Biotechnology, Gdansk, Poland). DNA was amplified with Mmp9_seq_1fw/1rev primer pair and amplicons were digested with HspaI (*Ksp*AI, Thermo Scientific, Waltham, MA, United States). In all assays Sanger sequencing-confirmed homozygous, heterozygous and wild-type DNA was used as controls.

Primers used in the study are shown in Table 1.

**TABLE 1:** Sequences of primers used in the study.

Name	Sequence	Description
Mmp9_UTRmut guide RNA	5′ TGCCCACCGTCCTTTCTTGT 3′	Guide RNA for CRISPR-based generation of mutation in *Mmp9* 3′ UTR
Mmp9_UTR_sgRNA_f	5′GAAATTAATACGACTCACTATAGGGtgcccaccgt cctttcttgtGTTTTAGAGCTAGAAATAGCAAGTTAAAATAAGGC 3′	Forward primer (inc. T7 Pol RNA promoter) for generation of sgRNA
Univ_IVsgRNA_rev	5′AAAAAGCACCGACTCGGTGCCACTTTTTCAAGTTGATA ACGGACTAgccttattttaacttgctatttctagctcta 3′	Reverse primer for construction of sgRNAs
Mmp9_UTRmut_O1	5′AGATAAGCTGATTGACTAAAGTAGCTGGAAAAGGTTGGG GATCCGTGTTTATTAGAAgtgagttAACAAGAAAGGACGGTGGGCA GAGAGAGCCCTGCCTGCCTCCACTCCTTCCCAGTC 3′	ODN donor for CRISPR-based generation of *Mmp9* UTRmut mice
Mmp9_seq_1fw	5′ GCGTGTGAGTTTCCAAAATG 3′	Forward sequencing primer for *Mmp9* UTRmut mice genotyping
Mmp9_seq_1rev	5′ TATTTATGCAGCGGTTGGAG 3′	Reverse sequencing primer for *Mmp9* UTRmut mice genotyping

### Nissl-staining

Mouse brains were fixed in 4% paraformaldehyde in PBS overnight at 4°C, then the brains were cryoprotected in 20% sucrose in PBS at 4°C for 48 h and frozen in −80°C. Next, the brains were cut coronally on cryostat (Cryostat Leica CM 1860) on 40-μm slices. Coronal sections were air dried on slides and stained with 0.1% cresyl violet solution (containing 3% acetic acid) for 5 min, washed, dehydrated, cleared in xylene, and coverslipped.

### RNA isolation and qRT-PCR

Hippocampi were dissected and frozen in −80°C. RNA was extracted using TRIzol (Thermo Fisher Scientific), DNA contamination was removed by 2 U of TURBO DNase (AM2238, Ambion, Austin, TX, United States) in the supplied buffer in 37°C for 30 min. Next, RNA was re-isolated with phenol/chloroform, precipitated with ethanol, and resuspended in 50 μl of RNase free water. Concentration was measured with Spectrophotometer DeNovix, Wilmington, DE, United States DS-11 at 260 nm.

Reverse transcription and qPCR of miR132 was performed using TaqMan MicroRNA Reverse Transcription Kit (4366596, Applied Biosystems), according to the manufacturer’s recommendations. RNA was diluted to 50 ng/μl and 250 ng was reverse transcribed using RT hsa-miR-132 primer (RT: 000457). Obtained products were diluted 2x and 2 μl of template was amplified using TaqMan MicroRNA Assays with has-miR-132 probe (TM: 000457) in a final reaction volume of 15 μl using Light Cycler 480 Probes Master Mix (Roche, Indianapolis, IN, United States) in a LightCycler480 (Roche).

To study the levels of *Mmp9* mRNA, RNA samples were reverse transcribed using random primers (GeneON, Ludwigshafen am Rhein, Germany; #S300; 200 ng/RT reaction) and SuperScript IV Reverse Transcriptase (Thermo Fisher Scientific). Next, the cDNA samples were amplified using custom sequence-specific primers and TaqMan MGB probes in a final reaction volume of 15 μl, using Light Cycler 480 Probes Master Mix (Roche) in a LightCycler480 (Roche).

Following TaqMan Gene Expression Assays (Thermo Fisher Scientific) were used: *Mmp9* (Mm00442991_m1), *Gapdh* (Mm99999915_g1), *Psd95* (Mm00492193_m1). Fold changes in expression were determined using the ΔΔ Ct (where Ct is the threshold cycle) relative quantification method. Values were normalized to the relative amounts of *Gapdh* mRNA.

### Gelatin zymography

Gelatin zymography was performed as previously described (Szklarczyk et al., 2002). Briefly, hippocampi were homogenized in buffer containing 10 mM CaCl_2_, 0.25% Triton X-100 and protease inhibitors (cOmplete EDTA-free Protease Inhibitor Cocktail, Roche). Homogenates were centrifuged at 6000 × *g* for 30 min at 4°C, the supernatant was removed and the pellet was resuspended in a buffer II (50 mM Tris, pH, 7.5; 0.1 mCaCl_2_, protease inhibitors), heated for 15 min at 60°C and then centrifuged at 10,000 × *g* for 30 min at 4°C. Supernatant was collected and stored in −80°C. Protein concentrations were measured using BCA protein assay (Thermo Fisher Scientific) and 30 μg of proteins were precipitated with 4 volumes of cold acetone overnight at −80°C. Next, the samples were centrifuged at 10,000 × g for 10 min at 4°C, the pellets were air-dried for 5 min at room temperature and resuspended in 20 μl of 2 × sample buffer in the absence of reducing reagents (Novex, Invitrogen, Carlsbad, CA, United States) and incubated for 30 min at 38°C with gentle shaking. Next, the samples were separated on 8% SDS-polyacrylamide gels containing 2 mg/ml gelatin. The gels were washed twice with 2.5% Triton X-100 for 30 min at room temperature and incubated in developing buffer (50 mM Tris-Cl, pH 7.5, 10 mM CaCl_2_, 1 μM ZnCl_2_, 1% Triton X-100, 0.02% NaN_3_) for 48 h at 37°C. Following staining with Coomassie brilliant blue, the gelatinolytic activities of Mmp9 and Mmp2 were detected as clear bands against a blue background and was quantified with ImageJ program. The intensity of Mmp9 bands was normalized to the Mmp2 bands.

### DiI staining of brain slices

To visualize morphology of dendritic spines 1,1’-dioctadecyl-3,3,3,3’-tetramethylindocarbocyanine perchlorate (DiI) staining was performed. Wild-type and *Mmp9* UTRmut mice at the age of 28 days (postnatal day 28, P28) were anesthetized with isoflurane and decapitated. The brains were quickly removed and both hemispheres were fixed with 4% paraformaldehyde in phosphate-buffered saline (PFA; 60 min at 4°C) and kept in cold PBS until further procedures were performed. Hemispheres were cut into 150 μm coronal slices with a vibratome (Leica VT 1000S). Random dendrite labeling was performed using 1.3 μm tungsten particles (Bio-Rad, Hercules, CA, United States) that were coated with propelled lipophilic fluorescent dye (DiI; Invitrogen, Carlsbad, CA, United States) that was delivered to the cells by gene gun (Bio-Rad) bombardment. Images of dendrites (50–200 μm) from the cell soma of the CA1 field of the hippocampus were acquired under 561 nm fluorescent illumination using a confocal microscope (Zeiss LSM 700) (63 × objective, 1.4 NA) at a pixel resolution of 1024 × 1024 with a 1.40 zoom, resulting in a 0.07 μm pixel size.

### Morphometric analysis of dendritic spines

The analysis of dendritic spine morphology was performed as described previously (Magnowska et al., 2016). The images that were acquired from the CA1 field of the hippocampus were processed using ImageJ software (National Institutes of Health, Bethesda, MD, United States) and analyzed semi-automatically using custom-written SpineMagick software^3^ (Ruszczycki et al., 2012). The analyzed dendritic spines belonged to secondary and ternary dendrites to reduce possible differences in spine morphology that are caused by the location of spines on dendrites with different ranks. The recorded parameters were the spine length, head width and the spine area. We also used a scale-free parameter (length/width ratio), which reflects spine shape. Dendritic segments of 4 animals per genotype were morphologically analyzed resulting in 67–79 images with 8890–9900 spines counted per experimental group. To determine spine density, approximately 4900–5000 μm of dendritic length was analyzed per genotype. Groups of dendritic spines were compared using nested unpaired Student’s *t*-test in case of spine morphology, in spine density analysis an unpaired Student’s *t*-test was used. The analysis was performed in a blinded fashion.

### Dendritic spine clustering

The virtual skeletons of dendritic spines were obtained in SpineMagick. Spine length was calculated as the length of the path from the spine top to the dendrite along the virtual skeleton of the spine. To analyze the shapes of spines, the virtual skeleton of each spine from an individual image was transformed to form a straight line. The images were then rescaled to normalize the spine area. For each spine diameter, we defined width as a function of distance from the dendrite, denoted d(h).

We classified 18,941 spines according to shape from WT and *Mmp9*UTRmut mice using a two-step procedure (Jasinska et al., 2016). First, all 18,941 d(h) functions were clustered into 36 clusters. Second, the clusters were manually sorted into three groups (i.e., mushroom, stubby, and long spines) based on visual inspection of clustered spines. The data analysis was performed using custom scripts that were written in Python. using NumPy and SciPy (Oliphant, 2007; Perez and Granger, 2007) and Matplotlib (Hunter, 2007).

### Electrophysiology

Mice were deeply anesthesized with isoflurane, decapitated and the brains were rapidly dissected and transfered into ice-cold cutting artificial cerebrospinal fluid (ACSF) consisting of (in mM): 87 NaCl, 2.5 KCl, 1.25 NaH_2_PO_4_, 25 NaHCO_3_, 0.5 CaCl_2_, 7 MgSO_4_, 20 D-glucose, 75 sacharose equilibrated with carbogen (5% CO_2_/95% O_2_). The brain was cut to two hemispheres and 350 μm thick coronal brain slices were cut in ice-cold cutting ACSF with Leica VT100S vibratome. Slices were then incubated for 15 min in cutting ACSF at 32°C. Next the slices were transferred to recording ACSF containing (in mM): 125 NaCl, 2.5 KCl, 1.25 NaH_2_PO_4_, 25 NaHCO_3_, 2.5 CaCl_2_, 1.5 MgSO_4_, 20 D-glucose equilibrated with carbogen and incubated for minimum 1 h at RT.

Extracellular field potential recordings were recorded in a submerged chamber perfused with recording ACSF in RT. The potentials were evoked with a custom built stimulus isolator with a concentric bipolar electrode (FHC, CBARC75) placed in the stRad of CA3. The stimulating pulses were delivered every 15 s and the pulse duration was 0.2 ms. The intensity of stimulation was adjusted to evoke 50% of maximal fEPSP. Recording electrodes (resistance 1–4 MΩ) were pulled from borosilicate glass (WPI, 1B120F-4) with a micropipette puller (Sutter Instruments, P-1000) and filled with recording ACSF. The recording electrode was placed in stRad of the dorsal CA1 area. Simultaneously, a second recording electrode was placed in the stPyr to measure population spikes. Recordings were acquired with MultiClamp 700B (Molecular Devices, CA, United States), Digidata 1550B (Molecular Devices, CA, United States) and Clampex 10.0 software (Molecular Devices, CA, United States). Input/output curves were obtained by increasing stimulation intensity by 25 μA in the range of 0–300 μA. The relative amplitude of fEPSP, population spikes and fiber volley were measured.

During LTP experiments a 20 min baseline was recorded. High frequency stimulation consisted of 1 train or 4 trains of 100 pulses at 100 Hz with 15 s intervals between consecutive trains. Then the recordings were carried out for another 60 min. For the sake of analysis 4 sweeps per minute were averaged. The results were normalized to average amplitude of fEPSP during baseline. The mean amplitude of fEPSP during 20 min baseline recording was compared with the mean amplitude of fEPSP during first 10 min after HFS (STP) and mean amplitude of fEPSP during the last 10 min of recording (LTP). Electrophysiology data was analyzed with AxoGraph 1.7.4 software (Axon Instruments, United States).

### Fear conditioning

A contextual fear conditioning paradigm was used to train the mice. Mice were trained in a Med Associates Inc. Fear Conditioning Chamber (St Albans, United States) connected to a computer running Video Freeze software. Mice were placed in the chamber on a metal grid platform and after 2.5 min of habituation, received three electric shocks (US, 2 s, 0.7 mA) with 90 s intervals. The animals were taken out from the experimental chamber 30 s after the last shock and put in the homecage. The training lasted 6 min between animals cage was cleaned with 70% ethanol solution and was readied for the next animal. The fear memory of the context, defined as a level of freezing in the context, was assessed for 5 min in the same experimental chamber 24 h and 1 week after training. All animals were trained, tested and sacrificed during the light phase of animals’ day (between 09.00 and 16.00 h). The training and testing times were counterbalanced between the groups.

### Preparation of synaptoneurosomes

Synaptoneurosomes were prepared as described previously (Scheetz et al., 2000; Dziembowska et al., 2012; Kuzniewska et al., 2018). Before tissue dissection Krebs buffer (2.5 mM CaCl_2_, 1.18 mM KH_2_PO_4_, 118.5 mM NaCl, 24.9 mM NaHCO_3_, 1.18 mM MgSO_4_, 3.8 mM MgCl_2_, 212.7 mM glucose) was aerated with an aquarium pump for 30 min at 4°C. Next, the pH was lowered to 7.4 using dry ice. The buffer was supplemented with 1 × protease inhibitor cocktail cOmplete EDTA-free (Roche) and 60 U/ml RNase Inhibitor (RiboLock, Thermo Fisher Scientific). Animals were euthanized by cervical dislocation, hippocampi and a part of cortex adjacent to the hippocampus were dissected. Tissue from one hemisphere (∼50 mg) was homogenized in 1.5 ml Krebs buffer using Dounce homogenizer with 10–12 strokes. All steps were kept ice-cold to prevent stimulation of synaptoneurosomes. Homogenates were loaded into 20 ml syringe and passed through a series of pre-soaked (with Krebs buffer) nylon mesh filters consecutively 100, 60, 30, and 10 μm (Merck Millipore, Kenilworth, NJ, United States) in cold room to 50 ml polypropylene tube, centrifuged at 1000 g for 15 min at 4°C, washed and the pellet was used for RNA-IP or frozen in −80°C for further RNA isolation.

### RNA coimmunoprecipitation (RNA-IP)

Immunoprecipitation was performed as previously described (Janusz et al., 2013), according to the modified protocol of Brown et al. (2001). Synaptoneurosomes from WT, *Mmp9* UTRmut and *Fmr1* KO mice (∼1600 μg of total protein) were resuspended in 1,200 μl of precipitation buffer (10 mm HEPES, pH 7.4, 400 mm NaCl, 30 mm EDTA, and 0.5% Triton X-100) supplemented with protease inhibitor cocktail (Sigma-Aldrich, St. Louis, MO, United States) and 100 U/ml RiboLock (Fermentas, Thermo Scientific, Waltham, MA, United States), then precleared with 120 μl of Dynabeads A for 2.5 h. Afterward, ∼50 μl of each supernatant was saved as input fraction for WB and RNA isolation. Precipitation was performed overnight in 4°C with 120 μl of antibody-bound Dynabeads Protein A, blocked beforehand with either anti-FMRP antibody (7G1–1-c) or normal mouse IgGs. Next, 1/6 of the beads was boiled with sample buffer for WB. From the remaining beads, total RNA was isolated with TRIzol (Invitrogen). For the quantitative real-time (qRT)-PCR, RNA was suspended in 11 μl of RNase free H2O Then the RNA was reverse-transcribed and the cDNA samples were amplified using custom sequence-specific primers and TaqMan MGB probes as described above. Fold changes were determined using the ΔΔ Ct (where Ct is the threshold cycle) relative quantification method. Values were normalized to the relative amounts of analyzed mRNA in IgG sample and compared to the abundance of mRNA in *Fmr1* KO samples.

### Quantification and statistical analysis

Unless otherwise noted, statistical analysis was performed using GraphPad Prism 7.0 (GraphPad Software, Inc.). Statistical details of experiments, including the statistical tests used and the value of n, are noted in figure legends.

## Results

### Knock-in mice with a mutation in miR132 binding site in *Mmp9* UTR does not display developmental abnormalities

**FIGURE 1:**
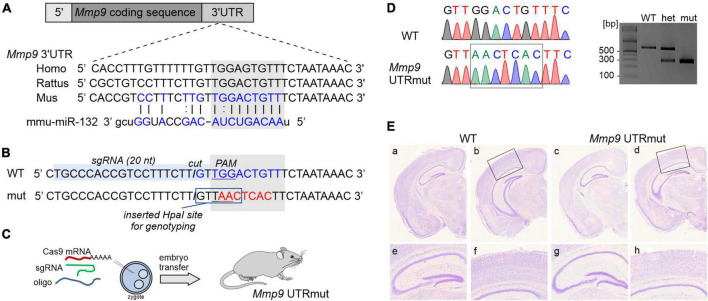
Generating mouse model harboring mutation in miR132 binding site in 3′UTR of *Mmp9* gene. **(A)** Schematic illustration of *Mmp9* allele, which contains coding sequence (dark gray) and untranslated regions (UTR, shown in light gray). The sequence of 3′UTR fragment that contains miR132 binding site is depicted in blue and aligned with human and rat sequences. Sequence of miR132 is aligned with its binding site. **(B)** Sequence of *Mmp9* 3′UTR fragment. The position of miR132-binding site (seed match) is marked with a gray box (WT seed match in blue and mutated in red). Position of the single-guide RNA (sgRNA) target is indicated with light-blue box. PAM, protospacer adjacent motif and the “cut” position is marked. To facilitate the detection of mutations, restriction site for *Hpa*I was introduced. **(C)** Schematic illustration of the strategy to introduce a knock-in mutations into the *Mmp9* locus using CRISPR/Cas9 strategy. **(D)** Alignment of chromatograms covering miR132 seed match site in *Mmp9*: wild-type (WT, top) and mutant (*Mmp9* UTRmut, bottom). Right panel, genotyping results of wild-type, heterozygous, and mutant mice based on *Hpa*I (*Ksp*AI) digestion of PCR amplicons covering miR132 seed match site in *Mmp9.* **(E)** Nissl-stained coronal sections of brains from wild-type (a, b, e, f) and mutant (c, d, g, h) mice. No neuroanatomical differences were observed in the WT and *Mmp9* UTRmut brains (a–d). Also, no abnormalities were found within the hippocampus (e, g) or in the cortex (f, h) of *Mmp9* UTRmut mice.

To dissect the effect of miR132 interaction with a single target site in *Mmp9* mRNA, we generated a mouse with a mutation in the 3′UTR of *Mmp9* gene using genome editing technology. A new knock-in mouse with a disrupted miR132 binding site in *Mmp9* mRNA 3′UTR was named “*Mmp9* UTRmut” (Figures 1A–D). The homozygous *Mmp9* UTRmut mice did not present any visibly harmful phenotype, were viable, fertile, gave similar litter sizes, and their survival was similar to WT mice. To verify whether the introduced mutation affects brain development, we looked at the general brain histology using the Nissl staining on coronal brain sections of wild-type (WT) and *Mmp9* UTRmut mice at postnatal day 32 (P32) (Figure 1E). Compared to their WT littermates, no alterations of overall brain morphology or individual brain structures, particularly hippocampus and cortex, were observed in *Mmp9* UTRmut animals.

### Mutated miR132 binding site in the *Mmp9* UTR did not affect mRNA levels and interactions with FMRP during hippocampus development

**FIGURE 2:**
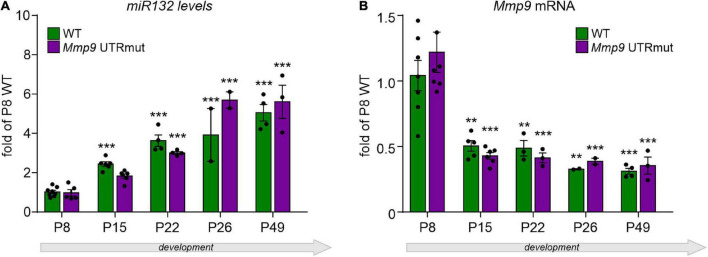
Relative expression of mature miR132 **(A)** and *Mmp9* mRNA **(B)** in the course of hippocampal development in *Mmp9* UTRmut and WT mice assessed by qRT-PCR. Generally, no significant differences in miR132 **(A)** and *Mmp9* mRNA **(B)** levels were observed among the two genotypes at any developmental stage. A two-way ANOVA revealed that there was statistically significant interaction between the effects of genotype and developmental stage on miR132 levels [**(A)** *F*_(4,34)_ = 3.61; *p* = 0.015], but not on Mmp9 mRNA levels [**(B)** *F*_(4,32)_ = 0.59; *p* = 0.68] in the hippocampus. Simple main effects analysis showed that genotype did not have a statistically significant effect neither on miR132 levels [*F*_(1,34)_ = 1.05; *p* = 0.31], nor on Mmp9 mRNA levels [*F*_(1,32)_ = 0.12; *p* = 0.74]. However, developmental stage did have a statistically significant effect on both miR132 [*F*_(4,34)_ = 69.53; *p* < 0.0001] and on Mmp9 mRNA [*F*_(4,32)_ = 23.35; *p* < 0.0001] levels. **(A)** Expression of miR132 was increased gradually and significantly during the development of the hippocampus at P15, P22, and P26 till adulthood (P49) in both genotypes (as compared to P8; ***p < 0.001; *post hoc* Sidak’s multiple comparisons test). **(B)** *Mmp9* mRNA levels were reduced by half already at P15 hippocampus and stayed on the same low level till adulthood (as compared to P8; **p < 0.01, ***p < 0.001; *post hoc* Sidak’s multiple comparisons test). Data is presented as mean ± SEM, normalized to P8 WT levels, dots correspond to single animals. *Mmp9* mRNA level was normalized to *Gapdh* mRNA.

Since the expression of both miR132 and *Mmp9* genes is induced by neuronal activity (Nudelman et al., 2010), we decided to elucidate their levels in the developing hippocampus of *Mmp9* UTRmut and wild-type mice. In the hippocampus, synaptic connections start to be established around birth with the extensive growth of axons and dendrites during the first two postnatal weeks and intensive synaptogenesis leading to adult patterns and integrative functions by the end of the first postnatal month (young adolescent mice) (Ben-Ari, 2001). Therefore, we evaluated the miR132 and *Mmp9* mRNA expression levels in the developing hippocampus of *Mmp9* UTRmut and WT mice at several postnatal stages: P8, P15, P22, P26, and P49, using qRT-PCR. We discovered that the expression of miR132 was induced in the second week of postnatal development and gradually increased till adulthood in both genotypes (Figure 2A). *Mmp9* mRNA expression analyzed in the same samples was highest at postnatal day eight (P8) and decreased already at P15 until adulthood in both *Mmp9* UTRmut and wild-type mice (Figure 2B).

Altogether, we found that the level of miR132 expression increases in the hippocampus development by 6 times when comparing P8 and P49. Contrary to miR132, the *Mmp9* mRNA level was decreased in the development of hippocampus. No significant differences were observed between the two studied genotypes suggesting that miR132 does not induce *Mmp9* mRNA degradation.

Our previous work (Jasinska et al., 2016) revealed that miR132 interacts with *Mmp9* mRNA. Also, we and others have shown that both *Mmp9* mRNA and miR132 associate with Fragile X mental retardation protein–FMRP (Edbauer et al., 2010; Janusz et al., 2013), an RNA binding protein primarily characterized for its role in regulating synaptic protein synthesis (Bassell and Warren, 2008; Darnell and Klann, 2013). FMRP can bind to G-rich sequences in RNA, and such motif in *Mmp9* 3′UTR is located in the proximity to the miR132 seed match site (Supplementary Figure 1A). We tested the hypothesis that the reversible repression of *Mmp9* mRNA translation by miR132 is regulated by FMRP protein. In order to determine whether disruption of miR132 seed match sequence in *Mmp9* 3′UTR affects the association of *Mmp9* mRNA with FMRP, we performed RNA coimmunoprecipitation on synaptoneurosomes isolated from the cerebral cortex and hippocampi of WT, Mmp9 UTRmut and Fmr1 KO mice (Supplementary Figure 1B). As shown in Supplementary Figure 1C, FMRP was immunoprecipitated by the 7G1-1 anti-FMRP antibody from WT and *Mmp9* UTRmut synaptoneurosomes, while it was not detected in the *Fmr1* KO immunoprecipitates (IP). Next, we performed qRT-PCR on immunoprecipitated fractions to assess levels of *Mmp9* mRNA and control mRNAs bound to FMRP. As previously reported, *Mmp9* mRNA (Janusz et al., 2013) as well as *Psd95* mRNA, a known target of FMRP (Muddashetty et al., 2007; Zalfa et al., 2007), were significantly enriched in the IPs from the WT mice compared with the *Fmr1* KO (Supplementary Figures 1D,E). However, we did not observe any significant differences in the levels of *Mmp9* mRNA in *Mmp9* UTRmut immunoprecipitates as compared to WT (Supplementary Figure 1D). Taken together, our data suggest that the mutation in the miR132 target site of *Mmp9* 3′UTR does not alter FMRP-*Mmp9* mRNA interaction. The levels of *Gapdh* mRNA, used as a control, did not differ between any IP fractions (Supplementary Figure 1F). Also, total mRNA levels of *Mmp9* and *Psd95* mRNAs were not significantly different between WT, *Mmp9* UTRmut and *Fmr1* KO synaptoneurosomes (Supplementary Figures 1G,H).

### Upregulation of Mmp9 protein in young adolescent *Mmp9* UTRmut mice correlates with altered structural plasticity of dendritic spines

**FIGURE 3:**
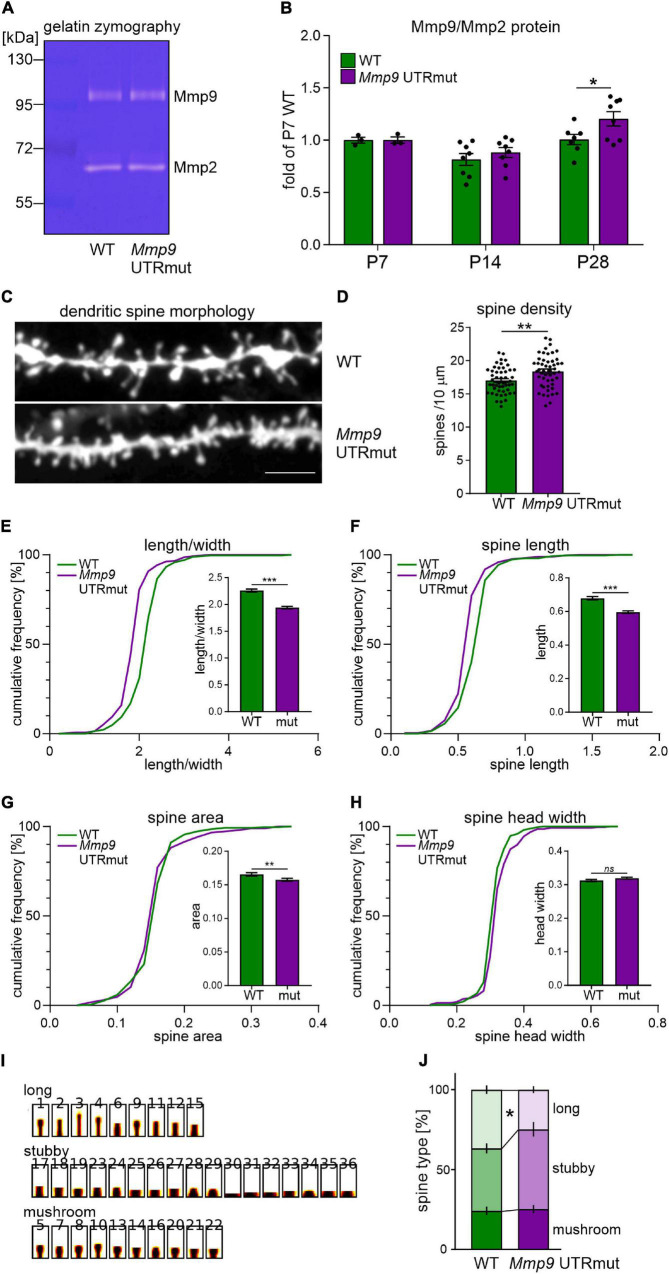
Mmp9 UTRmut mice display increased Mmp9 protein levels in the hippocampus and have shorter and more dense dendritic spines in the CA1 area. **(A)** Representative zymography gel revealing enzymatic activity of gelatinases from mouse hippocampus. Mmp9 and Mmp2 were identified as bright bands at ∼95 kDa and ∼65 kDa, respectively. **(B)** Two-way ANOVA did not reveal significant interaction between “genotype” and “development” factors [*F*_(2,31)_ = 1.23; *p* = 0.3063], however, “development” factor had significant effect [*F*_(2,31)_ = 11.62; *p* = 0.0002]. A significant increase of Mmp9 protein level was observed in the hippocampus of *Mmp9* UTRmut mice at P28 (*post hoc* Sidak’s multiple comparisons test; *p = 0.0444; *post hoc* Bonferroni’s multiple comparisons test; *p = 0.0450). Data is presented as mean ± SEM. Mmp9 levels were normalized to Mmp2 levels and presented as relative to WT P7. Dots correspond to single animals. **(C)** Examples of DiI-stained dendrites in the CA1 area of the hippocampus of wild-type (WT) and Mmp9 UTRmut mice. **(D)** Increased dendritic spine density in *Mmp9* UTRmut mice as compared to WT (unpaired Student’s *t*-test; ^**^p = 0.0022). Data is presented as mean ± SEM. Dots correspond to mean density from single image, *n* = 50 images/genotype; dendritic segments of four animals per genotype were analyzed. **(E–H)** Morphology of dendritic spines is altered in *Mmp9* UTRmut mice. Data is presented as cumulative frequency of analyzed parameters and as mean ± SEM. Significant differences in the spine shape parameter: decreased length/width [**(E)** ^***^p < 0.0001], spine length [**(F)** ^***^p < 0.0001] and spine area [**(G)** ^**^p = 0.0057] were found in *Mmp9* UTRmut mice as compared to wild-types. Spine head width was unchanged in *Mmp9* UTRmut mice [**(H)** *ns p* = 0.096]. Nested unpaired Student’s *t*-test was performed (dendritic segments of four animals per genotype were analyzed resulting in 67–79 images with 8890–9900 spines per experimental group). The analysis was performed in a blinded fashion. **(I,J)** Spines were clustered into three categories: long, stubby, and mushroom. *Mmp9* UTRmut mice exhibited a significant decrease in the population of long spines as compared with WT mice (*p = 0.0399; two-way ANOVA, *post hoc* Sidak’s multiple comparisons test).

MicroRNA fine-tuning of their target mRNAs may occur at the level of translational regulation. Since we did not observe any apparent alterations in *Mmp9* mRNA levels in *Mmp9* UTRmut mice, as a next step, we analyzed the level of Mmp9 protein in the hippocampus at various developmental stages. The level of Mmp9 protein in the brain is relatively low; therefore, it is poorly detectable with antibodies in western blotting. We used gel zymography, a proven and sensitive assay to quantify Mmp9 protein levels in the brain (Kleiner and Stetler-Stevenson, 1994; Snoek-van Beurden and Von den Hoff, 2005), which enables the visualization of Mmp2 and Mmp9 enzymatic activity in the polyacrylamide gel (Figure 3A).

We observed increased Mmp9 protein level in the hippocampus of young adolescent *Mmp9* UTRmut mice (postnatal day 28, P28; Figure 3B; *p* = 0.0444). In the juvenile mice (P7 and P14), no significant difference between genotypes was detected (Figure 3B).

MMP9 is secreted on dendritic spines in response to synaptic stimulation and has a well-documented effect on their structural plasticity (Wilczynski et al., 2008; Michaluk et al., 2011). We detected increased levels of MMP9 protein in the hippocampus of *Mmp9* UTRmut mice at P28, a developmental period of intense synapse formation and maturation. Therefore, we investigated the morphology and density of dendritic spines in the CA1 field of the hippocampus in the young adolescent (P28) *Mmp9* UTRmut and WT mice. Analysis was performed based on the DiI staining of coronal brain slices from four mice per genotype. Exemplary microphotographs of DiI stained dendrites are shown in Figure 3C. Using this approach, we demonstrated a significant increase in dendritic spine density in *Mmp9* UTRmut mice compared to WT (Figure 3D, *p* = 0.0022). Moreover, *Mmp9* UTRmut mice displayed changes in spine morphology. Dendritic spines in mutant mice had altered shape (Figure 3E, smaller spine length/head width ratio; *p* < 0.0001), were shorter (Figure 3F, smaller spine length; *p* < 0.0001), and had smaller spine area (Figure 3G; p = 0.0057). The spine head width remained unchanged (Figure 3H; *p* = 0.096). To further understand changes in dendritic spine shape, spines were clustered into three categories: long, stubby and mushroom. *Mmp9* UTRmut mice exhibited a significant decrease in the population of long spines as compared to WT mice (Figures 3I,J). Altogether in the hippocampus of *Mmp9* UTRmut mice, we observed shorter, however more dense dendritic spines, without a change in average spine head width, when compared to their wild-type littermates.

### *Mmp9* UTRmut mice show enhanced hippocampal long-term potentiation

The morphological changes in dendritic spine density and shape influence the electrophysiological properties of neurons. Having observed altered morphology and density of dendritic spines in the hippocampus of *Mmp9* UTRmut mice, we sought to determine its effect on electrophysiological activity of hippocampal circuits. Mmp9 is required for late-phase LTP stabilization in the hippocampus (Nagy et al., 2006). Interestingly, both Mmp9 knock-out mice, as well as rats with Mmp9 overexpression show decreased LTP in CA3-CA1 hippocampal projections (Nagy et al., 2006; Wiera et al., 2015; Magnowska et al., 2016), whereas transgenic mice overexpressing Mmp9 display prolonged LTP maintenance (Fragkouli et al., 2012). All this data indicate that balanced, fine-tuned Mmp9 levels are required for proper synaptic plasticity.

**FIGURE 4:**
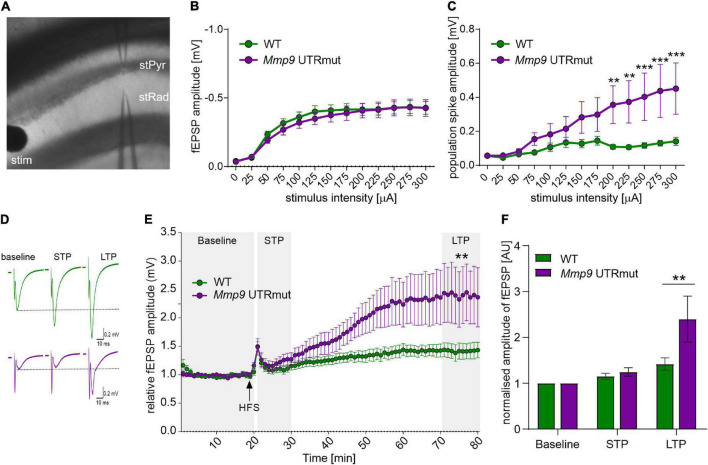
*Mmp9* UTRmut mice show enhanced hippocampal long-term potentiation. **(A)** Electrophysiological recordings setup depicting positions of stimulating (stim) and recording (rec1, rec2) electrodes in CA1 and CA3 fields of the hippocampus (stPyr–*stratum pyramidale*, stRad–*stratum radiatum*). **(B)** Input/output curve of fEPSP amplitude. Data is represented as mean ± SEM (n: WT 18 slices/5 mice, *Mmp9* UTRmut 18 slices/5 mice; two-way ANOVA with Bonferroni *post hoc* test). **(C)** Amplitude of population spikes measured in the CA1 *stratum pyramidale* during increasing intensity stimulation of Schaffer collaterals (n: WT-13 slices/5 mice, *Mmp9* UTRmut 9 slices/5 mice; two-way ANOVA with Bonferroni *post hoc* test; **p < 0.01, ***p < 0.001). **(D–F)** LTP induction at CA3–CA1 hippocampal synapses. High frequency stimulation (HFS) consisted of 4 trains of 100 pulses at 100 Hz with 15 s intervals between consecutive trains. Data is represented as mean of fEPSP amplitudes normalized to mean of baseline fEPSP amplitude ± SEM (n: WT 9 slices/5 mice, *Mmp9* UTRmut 9 slices/5 mice; repeated-measures two-way ANOVA with Bonferroni *post hoc* test; **p < 0.01). **(F)** Bar graph of the means ± SEM of normalized fEPSP amplitude calculated from the data in panel **(E)**. Averaged amplitudes of fEPSP during 20 min baseline, 10 min after HFS (STP), and last 10 min of the recording (LTP) were plotted (n: WT 9 slices/5 mice, *Mmp9* UTRmut 9 slices/5 mice; repeated-measures two-way ANOVA with Bonferroni *post hoc* test; **p < 0.01).

To examine the effect of mutation introduced in the non-coding region of *Mmp9* mRNA on the synaptic function, we measured basal synaptic transmission and synaptic plasticity at CA1 stratum radiatum synapses while stimulating Shaffer collaterals from CA3 hippocampal area, in acute brain slices from *Mmp9* UTRmut and wild-type mice (Figure 4A). Basal synaptic transmission in CA1 neurons, determined as the amplitude of fEPSP over a stimulation range 25–300 μA, was similar in slices from *Mmp9* UTRmut and WT mice (Figure 4B). Next, we induced LTP by high-frequency stimulation (HFS, 4 trains of 100 pulses at 100 Hz). This type of synaptic plasticity is long-lasting and was shown to be protein synthesis dependent (Frey et al., 1988; Impey et al., 1996). We recorded fEPSP for the next 60 min. The mean amplitude of fEPSP during the first 10 min after HFS, which corresponds to the short-term plasticity induced by stimulation (STP) was similar in both analyzed genotypes (Figures 4C,D). However, the mean amplitude of fEPSP during the last 10 min of recording, which corresponds to long-term plasticity (LTP) was significantly induced in both wild type and *Mmp9* UTRmut mice. Notably, the LTP was strongly enhanced in *Mmp9* UTRmut hippocampal slices, as compared to WT slices (Figures 4C–E). We also examined the LTP evoked by a weaker stimulus (1 × 100 Hz) in *Mmp9* UTRmut and WT mice hippocampi (Supplementary Figure 2). This weak protocol produces a synaptic potentiation that is shorter and not protein synthesis dependent (Frey et al., 1993). There was no significant difference between LTP recorded from WT and *Mmp9* UTRmut animals.

### Dysregulated specific targeting of miR132 to *Mmp9* mRNA enhances fear memory in mice

**FIGURE 5:**
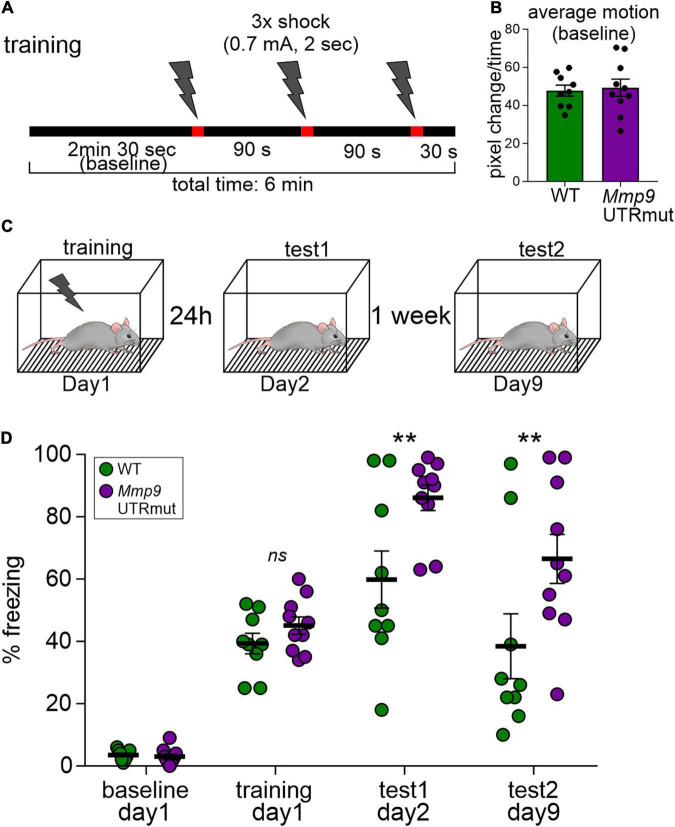
Learning is enhanced in contextual fear conditioning in the Mmp9 UTRmut mice. **(A)** Training protocol for fear conditioning indicating the number and timing of footshocks (lightning bolts). **(B)** Mouse activity within the conditioning chamber before the experiments was registered and quantified as an index of motion. **(C)** Scheme showing the experimental design. **(D)** Mice were conditioned according to the protocol and their freezing was recorded before the training–“baseline,” during the “training” and 24 h later–“test1.” Seven days after the training, the mice were exposed to the experimental chamber again and their freezing was recorded (“test2”). *Mmp9* UTRmut mice showed enhanced freezing in the test1 as compared to WT (^**^p = 0.0099). After 1 week *Mmp9* UTRmut mice still displayed enhanced freezing levels (^**^ *p* = 0.0051). Data is presented as mean ± SEM. Dots correspond to single animals. Repeated-measures two-way ANOVA, *post hoc* Sidak’s multiple comparisons test.

We demonstrated that blocking miR132-*Mmp9* mRNA interaction *in vivo* is sufficient to modulate Mmp9 protein levels in the developing brain and influence synaptic and structural plasticity in the hippocampus. With this in mind, we aimed at establishing the consequences of such dysregulation on mice behavior. Given the importance of both miR132 and Mmp9 in learning and memory formation, we verified whether these processes are impaired in *Mmp9* UTRmut mice. We tested the mice in the hippocampus-dependent contextual fear-conditioning task, in which animals learn to associate novel context (experimental chamber) with an aversive stimulus (electrical foot shock). The mice were trained with three-foot shocks and subsequently tested for contextual long-term fear memory at 24 h and 7 days after training (Figure 5A). The memory was scored as a freezing response to the context. Using this approach we found that *Mmp9* UTRmut mice showed improved contextual fear memory as demonstrated by enhanced freezing when exposed to the experimental chamber at 24 h and 7 days after the training (Figure 5B).

## Discussion

In the present study, we show for the first time that the dysregulated specific targeting of miR132 to *Mmp9* mRNA in the mouse brain *in vivo* results in the increased level of Mmp9 protein, which affects the function and structure of glutamatergic synapses and has an effect on mice memory formation. Our data points at the importance of complex and precise regulation of Mmp9 level by miR132 in the brain.

There is growing evidence to suggest that miRNAs are involved in social and anxiety-related behaviors and are dysregulated in the pathophysiology of human neuropsychiatric and neurodevelopmental disorders [Konopka et al., 2010 and reviewed by Smith and Kenny (2018) and Narayanan and Schratt (2020)]. However, so far the data implicating the role of miRNAs in animal behavior was obtained in miRNA overexpressing or miRNA knock-out mouse models. As miRNAs regulate many target mRNAs simultaneously, miRNA up- or downregulation results in modulation of entire cellular pathways. Therefore, although such functional studies are valuable to investigate the potential role of selected miRNA in the context of the whole organism, they do not allow for the examination of specific miRNA-target interaction. Furthermore, the role of miRNAs in human neurological disorders was based on miRNA profiling studies or genetic association studies, and the results are mainly correlative. Here we show for the first time a functional study of selected, specific miRNA-target interaction in the context of brain physiology and animal behavior. So far, attempts to create mouse models with disrupted seed-match sequences in 3′UTRs of selected genes were sparse (Dorsett et al., 2008; Lu et al., 2015; Mildner et al., 2017), and were never used to evaluate the effect of miRNA-target interaction on animal behavior. In the present study we created a unique mouse model to study the effect of single microRNA-mRNA interaction. We show that an increased level of Mmp9 in the hippocampus resulting from lack of posttranscriptional regulation by miR132 affects the morphology of dendritic spines, synaptic plasticity and has an effect on memory formation in mice. The observed changes of steady-state Mmp9 protein levels were not very pronounced in the hippocampus in Mmp9 UTRmut mice, pointing to its fine-tuning mode of regulation.

As miRNAs interaction with their target mRNAs seed region can lead to degradation of their target mRNA or translational inhibition, we aimed to elucidate whether the ablation of miR132 seed match in *Mmp9* 3′UTR *in vivo* will affect *Mmp9* levels. MiR132 expression progressively increases during mouse postnatal brain development in the hippocampus, olfactory bulb, and striatum (Nudelman et al., 2010). In addition, robust miR132 upregulation was induced in the primary visual cortex by the eye-opening during the critical period of its plasticity (Mellios et al., 2011). In accordance with those data, we also observed a significant increase in miR132 expression during the postnatal development of hippocampus in wild type and *Mmp9* UTRmut mice (Figure 2A).

Mmp9 plays an essential role in the developmental plasticity of hippocampus (Lepeta and Kaczmarek, 2015), and tight regulation of Mmp9 activity is critical for its function (Reinhard et al., 2015; Beroun et al., 2019). Mmp9 expression is also neuronal activity-dependent and is tightly regulated during brain development. In the hippocampus *Mmp9* expression levels are highest during the first postnatal week and decrease in adulthood (Aujla and Huntley, 2014). A similar pattern of *Mmp9* expression was observed in course of hippocampal development of wild-type and *Mmp9* UTRmut mice (Figure 2B).

The function of miRNA is highly context-dependent and may vary from the robust mRNA degradation during brain development to the subtle local effects on mRNA translation during dendritic spines remodeling (Schratt et al., 2006). Here we show that disrupting miR132 target sequence in the 3′UTR of *Mmp9* mRNA does not influence *Mmp9* mRNA levels in the hippocampus, indicating that miR132 recruitment to *Mmp9* does not promote mRNA degradation (Figure 2). Nevertheless, we demonstrate upregulation of Mmp9 protein levels in the brain of *Mmp9* UTRmut mice at different stages of development (Figure 3). Statistically significant results were observed in the hippocampus at P28. Taken together, our data points toward a translational regulation of *Mmp9* mRNA by miR132, which results with elevated Mmp9 protein level in the hippocampus of adolescent mice. In physiological conditions, Mmp9 is expressed at low levels in the brain and is induced by neuronal activity, particularly strongly in pathological conditions such as epilepsy, stroke, TBA (Konopacki et al., 2007; Wilczynski et al., 2008; Pijet et al., 2018, 2019). Mmp9 expression and activity is tightly regulated at the level of transcription, mRNA dendritic translocation, and local translation as well as protein activation of the enzyme [reviewed by Dziembowska and Wlodarczyk (2012)]. Both miR132 and Mmp9 are transported to the synapses where they can act to regulate synaptic function. Our results show that *Mmp9* UTRmut mice display relatively small, however significant upregulation of Mmp9 levels in the hippocampus (Figure 3B). In physiological conditions Mmp9 is expressed at a very low level in the brain and is induced by neuronal activity. Similarly, miR132 is an experience-dependent microRNA, that is rapidly upregulated in conditions of the synaptic plasticity. Therefore, presumably the mode of miR132-*Mmp9* interaction is also activity dependent, transient and local, restricted to the synaptic compartment. However, the activity-dependent regulation of *Mmp9* mRNA translation in *Mmp9* UTRmut mice needs to be addressed in the future. In this study the newly established mouse model *Mmp9* UTRmut mice allowed us to show that dysregulated specific targeting of miR132 to *Mmp9* mRNA has a functional relevance for the synaptic plasticity in the brain and can influence memory formation.

Dendritic spines are dynamic structures which exhibit morphological plasticity during brain development, learning and memory. Mmp9 is an extracellularly acting enzyme responsible for their structural plasticity (Michaluk et al., 2011; Szepesi et al., 2014). Similarly miR-132 can affect dendritic spine shape (Edbauer et al., 2010; Luikart et al., 2011; Mellios et al., 2011). Previously we have shown that miR-132-dependent regulation of *Mmp9* mRNA in neurons resulted in structural changes of dendritic spines. In the present study we observed more dense but shorter and smaller dendritic spines in the hippocampus of *Mmp9* UTRmut mice (Figure 3).

The morphological changes in dendritic spine density and shape was also shown to influence the electrophysiological properties of neurons (Tonnesen and Nagerl, 2016). In the CA1 field of *Mmp9* UTRmut mice hippocampus dendritic spines were smaller, but their head size did not differ from wild-type littermates, what can suggests that the average synapse strength is affected. However, more dense dendritic spines in *Mmp9* UTRmut mice point toward an increased number of synapses, implying enhanced complexity of neuronal circuits in the *Mmp9* UTRmut mice hippocampus. This hypothesis is supported by further data obtained in the current study, indicating that disrupting miR132-Mmp9 interaction *in vivo* has an apparent effect on long-term potentiation, explicitly enhancing its late phase (LTP) without significant changes of the basal synaptic strength (Figure 4). For the late phase of LTP the induction of gene expression and local protein synthesis is required and pharmacological inhibitors of translation block long-lasting forms of LTP without affecting early stages of LTP expression (Frey et al., 1988). In *Mmp9* UTRmut hippocampi only the long lasting LTP induced by 4 × 100 Hz stimulation was increased and not the early 1 × 100 HZ induced one, indicating translational regulation of MMP-9. Our data is consistent with previous reports showing that Mmp9 is required and upregulated during the late phase of LTP (Nagy et al., 2006; Huntley, 2012; Gorkiewicz et al., 2015). Pharmacological inhibition of Mmp9 blocks the induction of LTP and *Mmp9* knock-out mice display impaired, smaller LTP (Nagy et al., 2006). On the contrary, it was also shown that excessive Mmp9 activity in transgenic rats impairs both the induction and maintenance of LTP (Magnowska et al., 2016), pointing toward tight regulation of Mmp9 levels that are required for the proper synaptic transmission and plasticity.

Furthermore, we demonstrate that dysregulation of miR132 targeting to *Mmp9* mRNA has evident consequences for long-term memory. We show enhanced cognitive performance of *Mmp9* UTRmut mice in the fear-conditioning learning and memory task, without significant changes of mice activity before the training (Figure 5). Our data is in line with the previous reports describing impairments in a fear-conditioning memory task in Mmp9 knock-out mice (Nagy et al., 2006) and enhanced cognitive performance of Mmp9 overexpressing mice (Fragkouli et al., 2012). Both miR132 and Mmp9 were reported to be upregulated after fear conditioning (Ganguly et al., 2013; Wang et al., 2013). Consistently, knocking down miR132 in the hippocampus impaired the acquisition of fear memory in mice (Wang et al., 2013; Hansen et al., 2016). Altogether, we provide complementary evidence to the previous studies showing the involvement of both miR132 and Mmp9 in long-term hippocampus-dependent fear-conditioning memory tasks. We demonstrate that, above all, their interaction is pivotal in this learning paradigm.

In summary, we conclude that translational dysregulation of *Mmp9* mRNA expression by the lack of miR132 binding in the 3′UTR results in enhanced synaptic plasticity and influences memory formation.

## Data availability statement

The raw data supporting the conclusions of this article will be made available by the authors, without undue reservation.

## Ethics statement

The animal study was reviewed and approved by 1st Local Ethical Committee for Animal Experiments in Warsaw, Poland Pasteur St. 3 02-093 Warsaw, Poland.

## Author contributions

EB, OG, and JG developed the mouse model. BK, KRe, and JM collected tissue samples for the experiments. BK and KRe performed Nissl staining and gelatin zymography. BK extracted RNA, performed qRT-PCR analysis and IP experiments, analyzed dendritic spines, and prepared figures. MM prepared the DiI-stained brain slices and imaging. AN and AS performed the electrophysiological recordings. MZ performed the fear conditioning behavior. MD, AD, KRa, and BK designed experiments, analyzed the results, and wrote the manuscript. MD secured funding and supervised the project. All authors contributed to the article and approved the submitted version.

## Footnotes

1
www.crisprmice.eu

2
http://crispr.mit.edu

3
https://rdcu.be/cj5fb

## Supplementary material

Supplementary Figure 1 Mutation of miR132 binding site in 3′UTR of *Mmp9* gene does not affect FMRP-binding to *Mmp9* mRNA. **(A)** The sequence of mouse, rat, and human *Mmp9* 3′UTR fragment that contains miR132 binding site (seed) is marked with a gray box. MiR132 seed is depicted in blue and aligned with miR132 sequence. Position of G-quartet motif that forms quadraduplex structures and may be bound by FMRP is marked with a yellow box. **(B)** Scheme showing FMRP-*Mmp9* mRNA-miR132 complex. **(C–F)** RNA-immunoprecipitation using antibody on mouse synaptoneurosomal samples. **(C)** Western blot analysis of the immunoprecipitated FMRP from mouse synaptoneurosomes shows FMRP protein precipitated by the anti-FMRP 7G1-1 antibody in WT and *Mmp9* UTRmut mice. *Fmr1* KO mice and IgG IPs were used as negative controls. **(D–F)** qRT-PCR analysis of *Mmp9* mRNA, *Psd95* mRNA (positive control), and *Gapdh* mRNA (negative control) levels in immunoprecipitated samples from WT, *Mmp9* UTRmut, and *Fmr1* KO mice. **(D)** *Mmp9* mRNA associates with FMRP in synaptoneurosomes of WT (^**^*p* = 0.0057) and *Mmp9* UTRmut (^**^*p* = 0.0084) mice to the same extent, regardless of miR132 seed mutation. **(E)** As expected, *Psd95* mRNA also associates with FMRP in WT (^**^*p* = 0.0058) and *Mmp9* UTRmut (^**^*p* = 0.0046) synaptoneurosomes. **(F)** *Gapdh* mRNA does not associate with FMRP. Data is presented as mean ± SEM, *n* = 3 mice/genotype, One-way ANOVA, *post hoc* Dunnett’s multiple comparisons test. **(G,H)** *Mmp9* mRNA and *Psd95* mRNA levels in synaptoneurosomes isolated from WT and *Mmp9* UTRmut mice. *Gapdh* mRNA was used as endogenous control.

Supplementary Figure 2 *Mmp9* UTRmut mice show no significant differences in hippocampal early-LTP. **(A)** LTP induction at CA3–CA1 hippocampal synapses. High frequency stimulation (HFS) consisted of 1 train of 100 pulses at 100 Hz. Data is represented as mean of fEPSP amplitudes normalized to mean of baseline fEPSP amplitude ± SEM (WT, *n* = 6 slices; *Mmp9* UTRmut, *n* = 5 slices; repeated-measures two-way ANOVA with Bonferroni *post hoc* test; *p* > 0.8). **(B)** Bar graph of the means ± SEM of normalized fEPSP amplitude calculated from panel **(A)**. Averaged amplitudes of fEPSP during 20 min baseline, 10 min after HFS (STP), and last 10 min of the recording (LTP) were plotted (repeated-measures two-way ANOVA with Bonferroni *post hoc* test *p* = 0.0883).
