# Supplementary figures and images for: Disrupting interaction between miR-132 and Mmp9 3′UTR improves synaptic plasticity and memory in mice
Source: Front Mol Neurosci. 2022 Aug 5;15:924534. doi: 10.3389/fnmol.2022.924534 (PMC9389266; doi:10.3389/fnmol.2022.924534)

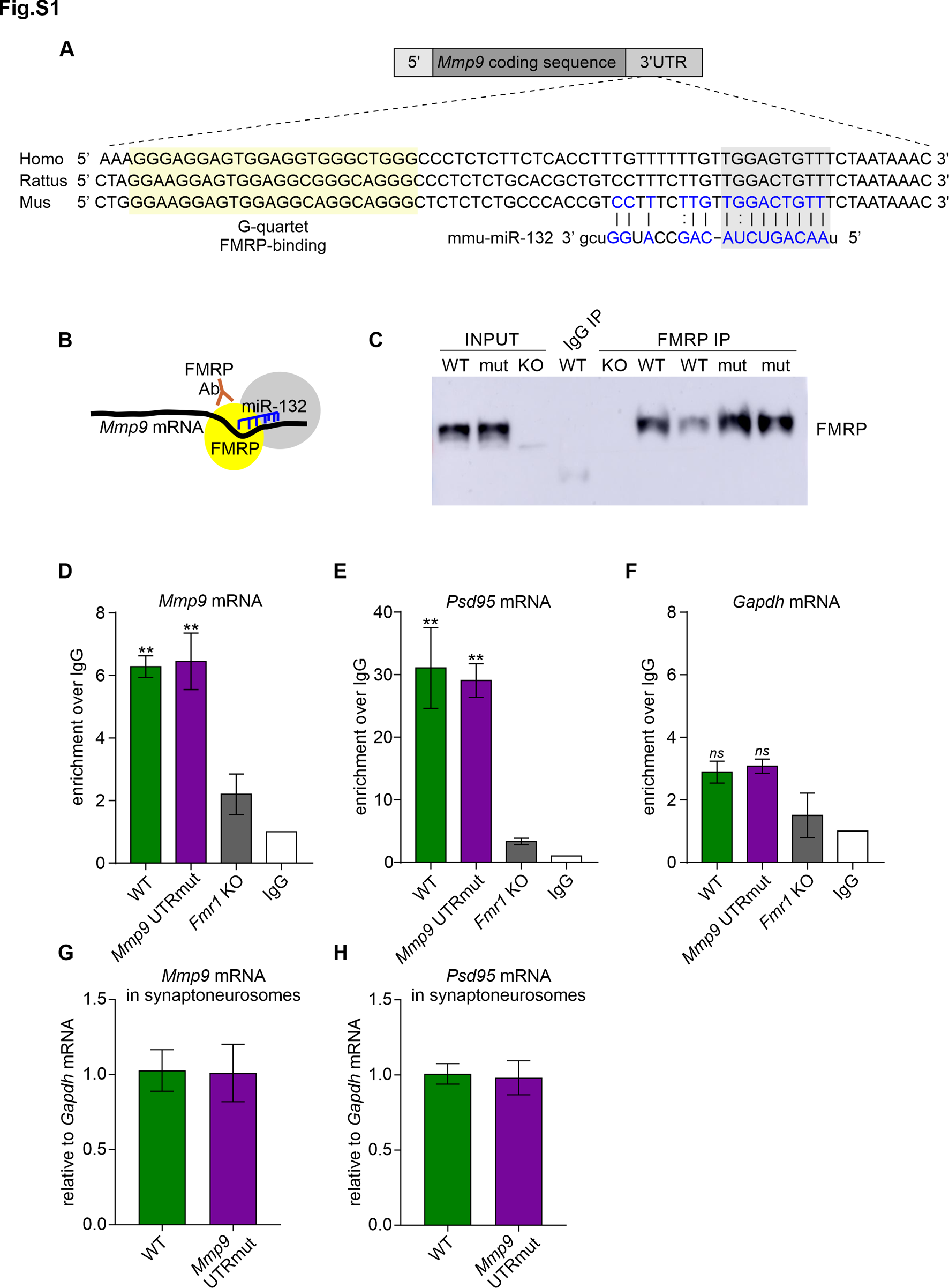

Supplement: Supplementary Figure 1 — Mutation of miR132 binding site in 3′UTR of Mmp9 gene does not affect FMRP-binding to Mmp9 mRNA. (A) The sequence of mouse, rat, and human Mmp9 3′UTR fragment that contains miR132 binding site (seed) is marked with a gray box. MiR132 seed is depicted in blue and aligned with miR132 sequence. Position of G-quartet motif that forms quadraduplex structures and may be bound by FMRP is marked with a yellow box. (B) Scheme showing FMRP-Mmp9 mRNA-miR132 complex. (C–F) RNA-immunoprecipitation using antibody on mouse synaptoneurosomal samples. (C) Western blot analysis of the immunoprecipitated FMRP from mouse synaptoneurosomes shows FMRP protein precipitated by the anti-FMRP 7G1-1 antibody in WT and Mmp9 UTRmut mice. Fmr1 KO mice and IgG IPs were used as negative controls. (D–F) qRT-PCR analysis of Mmp9 mRNA, Psd95 mRNA (positive control), and Gapdh mRNA (negative control) levels in immunoprecipitated samples from WT, Mmp9 UTRmut, and Fmr1 KO mice. (D) Mmp9 mRNA associates with FMRP in synaptoneurosomes of WT (**p = 0.0057) and Mmp9 UTRmut (**p = 0.0084) mice to the same extent, regardless of miR132 seed mutation. (E) As expected, Psd95 mRNA also associates with FMRP in WT (**p = 0.0058) and Mmp9 UTRmut (**p = 0.0046) synaptoneurosomes. (F) Gapdh mRNA does not associate with FMRP. Data is presented as mean ± SEM, n = 3 mice/genotype, One-way ANOVA, post hoc Dunnett’s multiple comparisons test. (G,H) Mmp9 mRNA and Psd95 mRNA levels in synaptoneurosomes isolated from WT and Mmp9 UTRmut mice. Gapdh mRNA was used as endogenous control. [file Image_1.TIF]

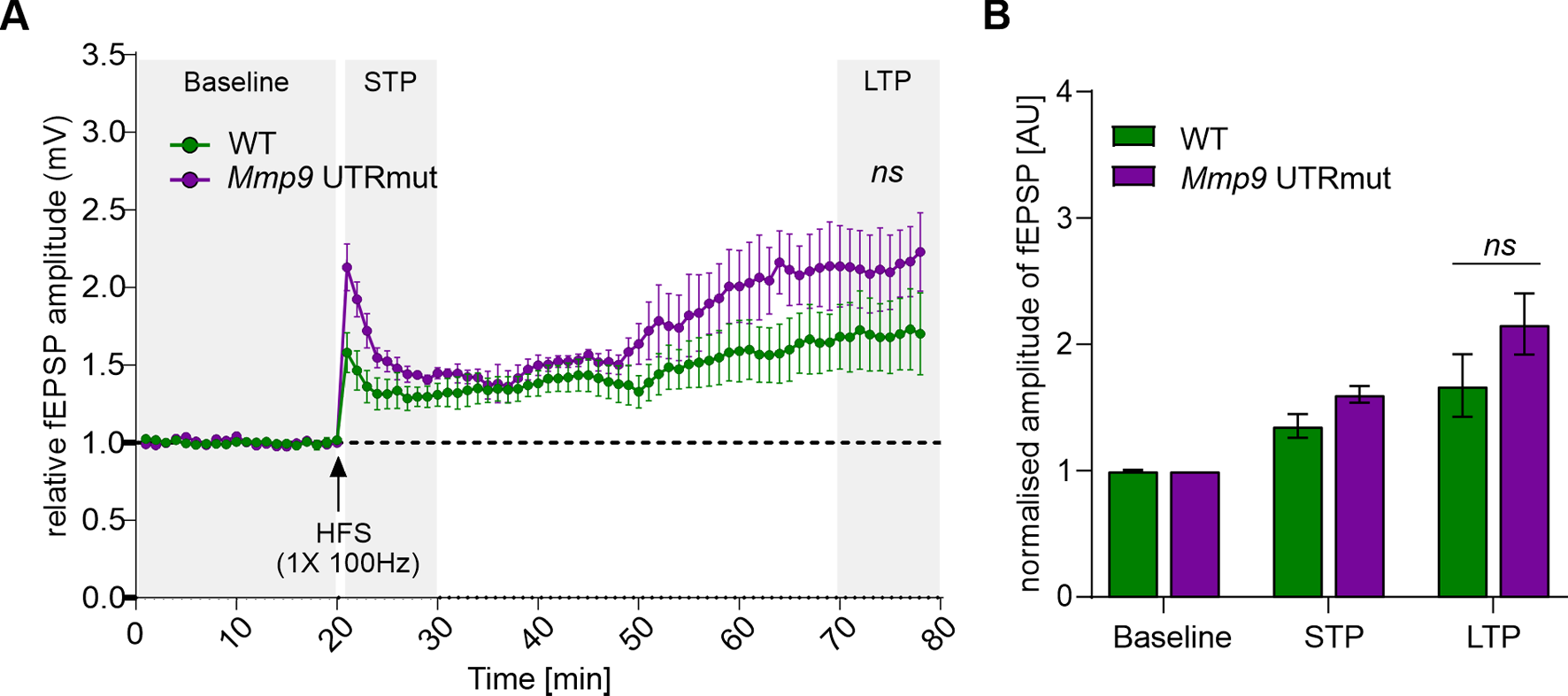

Supplement: Supplementary Figure 2 — Mmp9 UTRmut mice show no significant differences in hippocampal early-LTP. (A) LTP induction at CA3–CA1 hippocampal synapses. High frequency stimulation (HFS) consisted of 1 train of 100 pulses at 100 Hz. Data is represented as mean of fEPSP amplitudes normalized to mean of baseline fEPSP amplitude ± SEM (WT, n = 6 slices; Mmp9 UTRmut, n = 5 slices; repeated-measures two-way ANOVA with Bonferroni post hoc test; p > 0.8). (B) Bar graph of the means ± SEM of normalized fEPSP amplitude calculated from panel (A). Averaged amplitudes of fEPSP during 20 min baseline, 10 min after HFS (STP), and last 10 min of the recording (LTP) were plotted (repeated-measures two-way ANOVA with Bonferroni post hoc test p = 0.0883). [file Image_2.TIF]
